# WebScipio: reconstructing alternative splice variants of eukaryotic proteins

**DOI:** 10.1093/nar/gkt398

**Published:** 2013-05-15

**Authors:** Klas Hatje, Björn Hammesfahr, Martin Kollmar

**Affiliations:** Group Systems Biology of Motor Proteins, Department of NMR-based Structural Biology, Max-Planck-Institute for Biophysical Chemistry, Göttingen 37077, Germany

## Abstract

Accurate exon–intron structures are essential prerequisites in genomics, proteomics and for many protein family and single gene studies. We originally developed Scipio and the corresponding web service WebScipio for the reconstruction of gene structures based on protein sequences and available genome assemblies. WebScipio also allows predicting mutually exclusive spliced exons and tandemly arrayed gene duplicates. The obtained gene structures are illustrated in graphical schemes and can be analysed down to the nucleotide level. The set of eukaryotic genomes available at the WebScipio server is updated on a daily basis. The current version of the web server provides access to ∼3400 genome assembly files of >1100 sequenced eukaryotic species. Here, we have also extended the functionality by adding a module with which expressed sequence tag (EST) and cDNA data can be mapped to the reconstructed gene structure for the identification of all types of alternative splice variants. WebScipio has a user-friendly web interface, and we believe that the improved web server will provide better service to biologists interested in the gene structure corresponding to their protein of interest, including all types of alternative splice forms and tandem gene duplicates. WebScipio is freely available at http://www.webscipio.org.

## INTRODUCTION

Today, newly sequenced eukaryotic genomes become available almost daily (1,2). However, gene annotations are missing for most of them. Whole-genome annotations are usually done by using *ab initio* gene prediction software like AUGUSTUS (3), Fgenesh (4), GENSCAN (5), TWINSCAN (6), GeneMark (7) and mGene (8). *Ab initio* gene predictions can considerably be improved by incorporating additional data like EST/cDNA data sets, RNA-Seq data, curated protein annotations and genome alignments. Tools that combine these aspects are AUGUSTUS, Fgenesh+, GeneWise/Wise2 (9) and GenomeScan (10). Although these methods need species-specific sets of parameters for best performance, they can also be used to predict genes in any user-provided genomic sequences. However, automatic gene annotations are error prone and cannot handle sequencing and assembly errors leading to frame-shifts, in-frame stop codons and gaps in genes, but accurate gene structures and translations are needed in genomics, proteomics and in protein family and single-gene studies. In these cases, annotations can manually be improved, which is best done at the protein level (11), and gene structures obtained based on the revised protein sequences. The most convenient tools to map protein sequences onto genome sequences are Scipio (12), ProSplign, which is an integral part of NCBI’s Genome Annotation Pipeline (Gnomon) (13), Exonerate (14) and Prot_map (15). From the latter tools, Scipio is the only software, which is accessible through a web interface.

We have developed Scipio as a tool to determine the precise gene structure, given a protein and a genome sequence (12). To facilitate its usage for non-experts and to provide easy access to the available eukaryotic genome data, a web interface to Scipio has been developed, called WebScipio (16). Initially, Scipio and WebScipio were intended for correctly mapping a protein query onto the respective genome sequence, but soon it has been realized that the tool can also be used to identify and reconstruct protein homologues of the query protein within the same species and in closely related species (17). In this respect, WebScipio is currently limited to a sequence identity of ∼80%. WebScipio has also been extended to allow the prediction and identification of mutually exclusive spliced exons (MXEs) (18) and the determination of tandemly arrayed gene duplicates (19). Here, we report on new developments of WebScipio. The most important extension is an additional module allowing mapping the reconstructed gene against available cDNA/EST data to determine any type of alternative splicing. Although genome browsers offering pre-computed sets of mapped cDNA/EST data sets are available for several model organisms [e.g. at FlyBase (20) or the UCSC Genome Browser page (21)], the new WebScipio functionality should be useful for less annotated and analysed species.

## WEBSCIPIO

**Figure 1.**
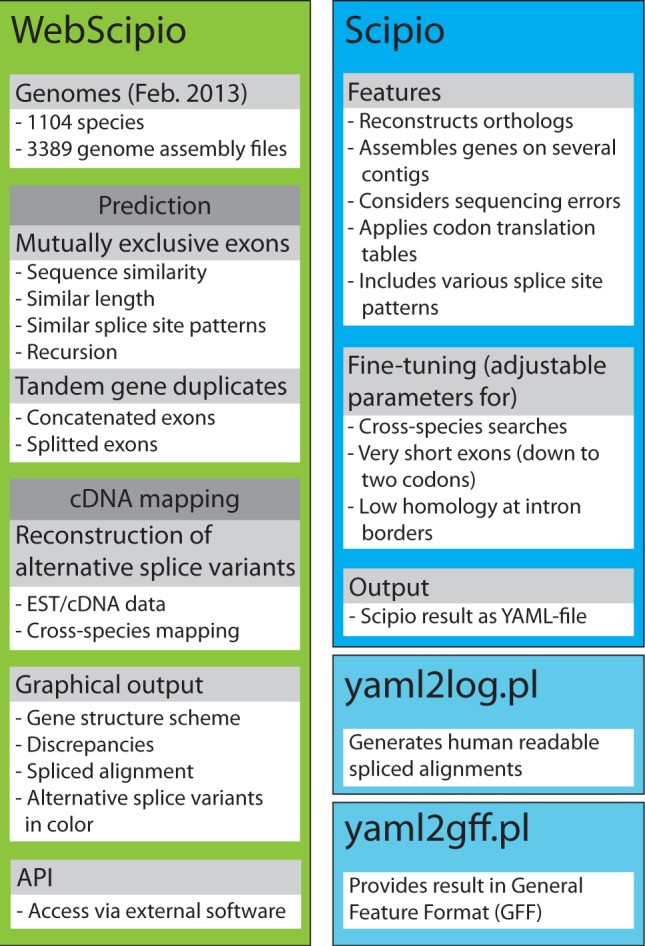
The scheme summarizes the most important features of Scipio and WebScipio. Scipio’s main task is the exact reconstruction of the gene structure of given protein sequences, but it can also be used to identify homologues within the same and in related species. WebScipio is the graphical interface to Scipio providing access to almost all sequenced eukaryotes. In addition, it allows the prediction of mutually exclusive exons and tandemly arrayed gene duplicates, and the reconstruction of all types of alternative splice variants based on cDNA/EST mapping.

The basic WebScipio architecture and work flow remains the same (Figure 1). It is build on a Ruby on Rails web application that executes a Scipio gene reconstruction and displays results back to the user. Reconstructions of alternative splice variants based on cDNA data and predictions of MXEs and tandemly arrayed gene duplicates are done with Ruby scripts based on the Scipio output. The web interface provides several examples representing the different types of possible gene reconstructions and predictions.

>

### Input (select a species, provide a protein sequence, adjust optional parameters)

**Figure 2.**
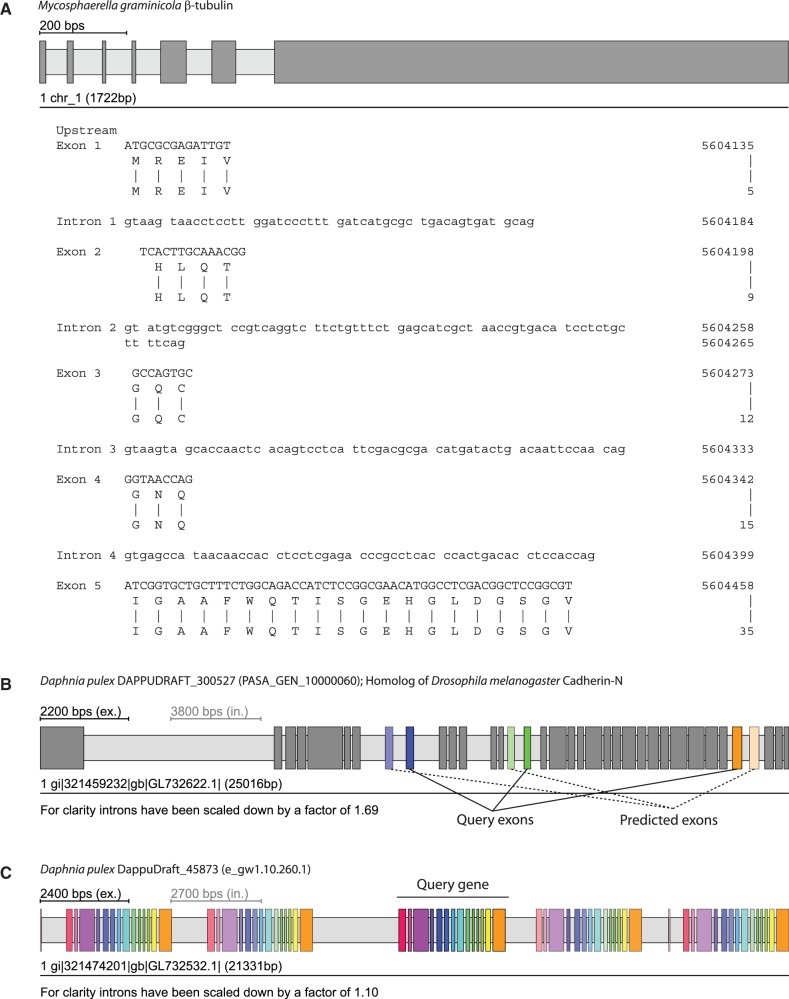
Examples for running WebScipio using previously implemented advanced options and prediction algorithms. Exons are represented as dark-grey or coloured bars, and introns as light-grey bars. (**A**) The N-termini of the β-tubulin genes in fungi (here *Mycosphaerella graminicola*) are encoded by several very short exons. These exons are not recognized by *ab initio* gene prediction programs and hardly identified in other gene reconstruction software. (**B**) Predicted clusters of MXEs are displayed in colour. The exon of the cluster, which was present in the query sequence, is shown in full colour, whereas the predicted MXEs are shown with opacity that correlates to their similarity to the query exon (high similarity = high opacity). (**C**) Tandemly arrayed gene duplicates can be predicted within a user, given genomic region. Here, the query gene was the central gene of the cluster. In general, homologous exons in duplicated genes have the same colour as the exons of the query gene. Like with the MXE colouring, similarity to the query exon is expressed through opacity. Exceptions are exons whose homologues are fused or split exons in the duplicated gene.

WebScipio requires users to select a eukaryotic genome assembly and to provide protein sequences in plain or FASTA format. Alternatively, users might upload their own genome data (current limit: 1 MB). Subsequently, several advanced options are available: (i) Users can adjust the parameters for the Scipio run to obtain better results for difficult query/target sequences and for cross-species searches (17). Although the standard parameters should work in most cases, there are gene reconstructions needing adjustments like subsequent very short exons (Figure 2A), ambiguities between exonic and intronic sequences, low homology of certain regions in cross-species searches and usage of specific codon translation tables. (ii) WebScipio can predict MXE candidates for the exons of the reconstructed gene [Figure 2B; (18)]. The prediction parameters can be adjusted (length difference and similarity between MXEs of a cluster, optionally a recursive search). The options to search with all exons in all introns and to search the up- and downstream regions can help in detecting potentially *trans*-spliced genes and tandemly arrayed gene duplicates. (iii) WebScipio can be enabled to search for all types of alternative spliced coding exons by mapping cDNA data onto the reconstructed gene structure (for more details see later in the text). (iv) WebScipio can predict tandemly arrayed gene duplicates (19). In contrast to the superficial detection of these duplicates via options of the MXE search, this algorithm searches for duplicates on both strands of the up- and downstream DNA and allows finding fused and split exons in the gene duplicates compared with the query gene (Figure 2C).

>

### Output

WebScipio provides several options to inspect and download the results: (i) A graphical representation of the gene structure with detailed information and statistics. Exons and introns are displayed as grey boxes (Figure 2). Discrepancies between query sequence and target genome like mismatches, sequence shifts, in-frame stop codons and unmatched sequences are shown in specific colours at their respective positions (examples are provided on the web page). Clusters of mutually exclusive exons and homologous exons in tandemly arrayed gene duplicates are shown in same colours. (ii) An alignment of the protein query to the genomic DNA enabling the user to inspect the resulting gene structure down to the nucleotide level (Figure 2A). (iii) A detailed evaluation of the discrepancies between query protein sequence and target translation, which is especially useful in cross-species searches and searches for protein homologues. (iv) The results are available in various formats for storing and further processing. The raw data are provided as YAML Ain't Markup Language (YAML) file, which can be uploaded again for further analyses, and as General Feature Format (GFF) file, which is the standard output format of gene annotation software. All types of generated sequences [exons, introns, genomic DNA, coding DNA sequence (CDS), translation] can be downloaded separately. The gene structure schemes are available in the Scalable Vector Graphics format providing high-quality figures for publications.

## NEW DEVELOPMENTS

### Extended set of target species

One of WebScipio’s major goals is to provide access to reconstructing genes in all sequenced eukaryotes. As such, we update the list of available eukaryotes and genome assemblies almost on a daily basis based on the data provided by diArk (1,2). Compared with our last report (17), WebScipio now offers 3389 genome assembly files (2111 in February 2011) for 1104 eukaryotic species (592 in February 2011).

### Reconstructing alternative splice variants

The reconstruction of alternative splice variants is based on mapping of cDNA or EST data to an exon–intron gene structure reconstructed by Scipio. Either, a previously reconstructed gene structure can be uploaded (menu entry ‘Upload Result File’) or a genome assembly has to be selected and a protein sequence provided for a new reconstruction. In the options section ‘Search for Alternatively Spliced Exons’, the user first chooses one or more cDNA data sets by species in an auto-completion field (the species used for the gene reconstruction is pre-selected) and by type (EST or mRNA). The EST data have been derived from the NCBI dbEST and the cDNA data from the NCBI nr database (22). Both databases are checked for updates monthly. WebScipio also allows uploading own cDNA data in FASTA format. Subsequently, BLAT (23) search parameters can be adjusted.

The cDNA sequence mapping is done in two steps. First, all sequences of the selected cDNA data set are mapped against the CDS of the Scipio result using BLAT (23) to derive all matching cDNA sequences. Alternatively, the cDNA data can be mapped against the genomic DNA sequence, which is useful to check for those sequences that only map to introns (this option, however, considerably increases the run-time for long genes) and against the translated CDS, which is best suited in searches in which cDNA data sets from a species different from the query sequence are used. Many of the BLAT parameters can be adjusted via the web interface. In the second step, the obtained matching cDNA sequences are mapped against the genomic DNA sequence of the Scipio result. This second step is necessary because matching hits might contain sequence not present in the CDS of the Scipio result and allows to identify alternative splice forms.

**Figure 3.**
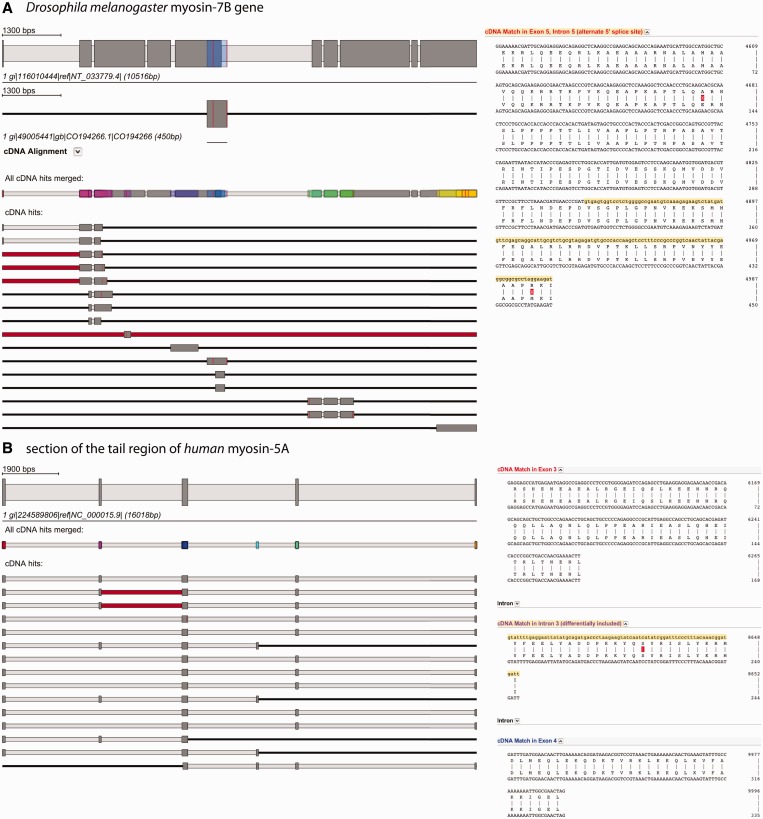
Alternative splice variants are detected by mapping EST/cDNA onto the reconstructed gene. (**A**) The example shows the search for alternative splice variants in the *Drosophila melanogaster* myosin-7B gene. The gene structure on top displays the reconstructed gene. All EST/cDNA clones mapping to the gene are listed below (red bars represent parts of the clones that do not map to the query gene, red lines represent single mismatches), and every of them can be activated by clicking for further inspection (second scheme from top). The position, where this specific clone is mapping onto the query gene, is shown in colour (blue bar on the myosin-7B gene structure). Always, a merged scheme is shown, in which all regions of the query gene, to which EST/cDNA clones mapped, are highlighted in colour (third scheme from top). This should provide the user an overview about the regions covered when dozens to hundreds EST/cDNA clones map. The spliced alignment on the right represents the section of the alignment result view that contains the suggested alternative splice variant and is displayed by clicking on ‘cDNA alignment’ below the scheme of this clone. (**B**) This example shows a section of the tail region of the human myosin-5A gene and a selection of EST clones mapping to it. The red bars in the second and third cDNA clone indicate that part of these clones could not be mapped to the query sequence. The alignment on the right side corresponds to the beginning of the first EST hit. Colours on exon descriptions correspond to the exon, where this part maps (same colour as in the merged EST scheme) and the alternative splice type as suggested by the mapped EST clones is given.

For displaying the results, BLAT sequence coordinates are subsequently converted into Scipio coordinates. Hits are shown as schemes below the exon–intron gene structure of the query sequence, displaying mapped EST/cDNA sequence as dark grey, intronic regions as light grey and unmatched EST/cDNA sequence as red bars (Figure 3). Mismatches between gene and EST/cDNA sequence are shown as red lines. The cDNA sequences of each hit are translated and aligned to the translation of the genomic sequence. These alignments can be displayed for each hit separately in the web interface. Potential alternative splice variants are present if cDNA hits not only map to the exons already included in the gene structure but also overlap with intronic regions. WebScipio distinguishes between alternative 5′ and 3′ splice sites, exon skipping (differentially included exons) and intron retention events, and the supposed splice variant is displayed as tooltip on the scheme of the hit and in the header of the alignment. The detailed flowchart of the cDNA mapping procedure is described in Supplementary Figure S1.

>

As examples for the usage of the new function, we present a case of an alternative 5′ splice site and a sequence containing a cluster of differentially included exons (Figure 3). Figure 3A illustrates the analysis of the myosin heavy chain gene 7B of *Drosophila melanogaster* (LOCUS). Sixteen EST sequences map to the gene covering ∼60% of the CDS. Three of the sequences show the extension of exon 5 into the subsequent intron region suggesting an alternative 5′ splice site for exon 5 and at least two isoforms for the *Drosophila* myosin-7B gene. In the second example (Figure 3B), the search for alternative splice variants for part of the tail region of human myosin-5A is shown. Because the entire myosin-5A gene is covered by hundreds of EST sequences, we focus on this specific part of the tail for simplicity. The cDNA data mapping supports the differential inclusion of exon 2 (purple coloured exon) and identified an additional differentially included exon that was not present in the query sequence (light-blue exon). Exon 2corresponds to ‘exon F’ of the alternatively spliced exons of the myosin-5A tail (24). The alignments of the EST sequences to the genomic DNA show two non-synonymous substitutions for *Drosophila* myosin-7B (Figure 3A) and one case of synonymous substitution for human myosin-5A (Figure 3B).

## CONCLUSIONS

Herein, we present an updated version of WebScipio, the web interface to the Scipio gene reconstruction software. WebScipio is unique in providing direct access to most of the sequenced eukaryotes whose number has doubled compared with the previous version. The number of genome assemblies now exceeds 3300 (January 2013). All types of alternative splice variants can be reconstructed based on cDNA/EST data mapping and a unique prediction algorithm for mutually exclusive exons.

## SUPPLEMENTARY DATA

Supplementary Data are available at NAR Online: Supplementary Figure 1.

## FUNDING

German Research Foundation [KO 2251/6-1 to M.K.]. Funding for open access charge: Max-Planck-Institute for Biophysical Chemistry.

*Conflict of interest statement.* None declared.

## References

[1] Odronitz F, Hellkamp M, Kollmar M (2007). diArk—a resource for eukaryotic genome research. BMC Genomics.

[2] Hammesfahr B, Odronitz F, Hellkamp M, Kollmar M (2011). diArk 2.0 provides detailed analyses of the ever increasing eukaryotic genome sequencing data. BMC Res. Notes.

[3] Stanke M, Waack S (2003). Gene prediction with a hidden Markov model and a new intron submodel. Bioinformatics.

[4] Salamov AA, Solovyev VV (2000). Ab initio gene finding in *Drosophila* genomic DNA. Genome Res..

[5] Burge C, Karlin S (1997). Prediction of complete gene structures in human genomic DNA. J. Mol. Biol..

[6] Van Baren MJ, Koebbe BC, Brent MR (2007). Using N-SCAN or TWINSCAN to predict gene structures in genomic DNA sequences. Curr. Protoc. Bioinformatics.

[7] Borodovsky M, Lomsadze A (2011). Eukaryotic gene prediction using GeneMark.hmm-E and GeneMark-ES. Curr. Protoc. Bioinformatics.

[8] Schweikert G, Zien A, Zeller G, Behr J, Dieterich C, Ong CS, Philips P, De Bona F, Hartmann L, Bohlen A (2009). mGene: accurate SVM-based gene finding with an application to nematode genomes. Genome Res..

[9] Birney E, Clamp M, Durbin R (2004). Genewise and genomewise. Genome Res..

[10] Yeh RF, Lim LP, Burge CB (2001). Computational inference of homologous gene structures in the human genome. Genome Res..

[11] Yandell M, Ence D (2012). A beginner’s guide to eukaryotic genome annotation. Nat. Rev. Genet..

[12] Keller O, Odronitz F, Stanke M, Kollmar M, Waack S (2008). Scipio: using protein sequences to determine the precise exon/intron structures of genes and their orthologs in closely related species. BMC Bioinformatics.

[13] Sayers EW, Barrett T, Benson DA, Bolton E, Bryant SH, Canese K, Chetvernin V, Church DM, DiCuccio M, Federhen S (2011). Database resources of the National Center for Biotechnology Information. Nucleic Acids Res..

[14] Slater GS, Birney E (2005). Automated generation of heuristics for biological sequence comparison. BMC Bioinformatics.

[15] Solovyev V, Kosarev P, Seledsov I, Vorobyev D (2006). Automatic annotation of eukaryotic genes, pseudogenes and promoters. Genome Biol..

[16] Odronitz F, Pillmann H, Keller O, Waack S, Kollmar M (2008). WebScipio: an online tool for the determination of gene structures using protein sequences. BMC Genomics.

[17] Hatje K, Keller O, Hammesfahr B, Pillmann H, Waack S, Kollmar M (2011). Cross-species protein sequence and gene structure prediction with fine-tuned Webscipio 2.0 and Scipio. BMC Res. Notes.

[18] Pillmann H, Hatje K, Odronitz F, Hammesfahr B, Kollmar M (2011). Predicting mutually exclusive spliced exons based on exon length, splice site and reading frame conservation, and exon sequence homology. BMC Bioinformatics.

[19] Hatje K, Kollmar M, Friedberg F (2011). Predicting tandemly arrayed gene duplicates with webscipio. Gene Duplication.

[20] Drysdale R (2008). FlyBase: a database for the Drosophila research community. Methods Mol. Biol..

[21] Meyer LR, Zweig AS, Hinrichs AS, Karolchik D, Kuhn RM, Wong M, Sloan CA, Rosenbloom KR, Roe G, Rhead B (2012). The UCSC genome browser database: extensions and updates 2013. Nucleic Acids Res..

[22] Benson DA, Cavanaugh M, Clark K, Karsch-Mizrachi I, Lipman DJ, Ostell J, Sayers EW (2013). GenBank. Nucleic Acids Res..

[23] Kent WJ (2002). BLAT—The BLAST-like alignment tool. Genome Res..

[24] Roland JT, Lapierre LA, Goldenring JR (2009). Alternative splicing in class V myosins determines association with Rab10. J. Biol. Chem..

